# Confounding factors of non-invasive tests for nonalcoholic fatty liver disease

**DOI:** 10.1007/s00535-020-01686-8

**Published:** 2020-05-25

**Authors:** Janae Wentong Wai, Charmaine Fu, Vincent Wai-Sun Wong

**Affiliations:** 1 Department of Medicine and Therapeutics, The Chinese University of Hong Kong, 9/F, Prince of Wales Hospital, 30-32 Ngan Shing Street, Hong Kong, China; 2 State Key Laboratory of Digestive Disease, The Chinese University of Hong Kong, Hong Kong, China

**Keywords:** Nonalcoholic fatty liver disease, Nonalcoholic steatohepatitis, Liver fibrosis, Cirrhosis, Transient elastography

## Abstract

Nonalcoholic fatty liver disease (NAFLD) affects at least 25% of the general adult population worldwide. Because only a fraction of the patients would develop liver-related complications, it is preferable to perform non-invasive tests as the initial assessment. This review summarizes the known and potential confounding factors that affect the performance of non-invasive tests of hepatic steatosis and fibrosis in patients with NAFLD. Clinicians may apply the knowledge and exercise caution in selecting investigations and interpreting test results when confounding factors are present.

## Introduction

Nonalcoholic fatty liver disease (NAFLD) affects at least 25% of the global adult population [1, 2], and has become one of the leading causes of cirrhosis and hepatocellular carcinoma in Western countries [3, 4]. Although NAFLD appears to be milder in Asia [5], the rise in liver decompensation, hepatocellular carcinoma and death due to NAFLD is particularly steep in China [6]. NAFLD is divided into nonalcoholic fatty liver (NAFL, also known as simple steatosis) and nonalcoholic steatohepatitis (NASH) according to disease severity [7]. The latter is characterized by the presence of lobular inflammation and hepatocyte ballooning. While NAFL can progress to NASH and vice versa [8], on the whole, NASH patients are at a higher risk of fibrosis progression and liver-related complications [9, 10]. Accumulating fibrosis eventually results in cirrhosis and subsequent complications. Because fibrosis is the path towards cirrhosis, it comes as no surprise that fibrosis has the strongest correlation with liver-related morbidity and mortality in longitudinal studies [11].

In the past, liver biopsy was the primary investigation to determine the severity of NAFLD. It also serves to exclude other liver diseases. However, it is an invasive procedure with a 0.3% risk of bleeding. Patient acceptability is low, and it is undesirable to perform liver biopsy repeatedly to assess disease progression and treatment response. More importantly, liver biopsy is not a real gold standard for the evaluation of the histological features of NAFLD. Because a liver biopsy sample only represents around 1/50,000 of the entire liver volume, there is considerable sampling variability [12]. The interpretation of individual histological features of NAFLD also suffers from intraobserver and interobserver variability [13]. Among the key histological features of NAFLD, the reproducibility and interobserver concordance are particularly poor for lobular inflammation and hepatocyte ballooning, which are both defining features of NASH. There has therefore been much interest in the development of non-invasive tests to replace or supplement liver biopsy in the past 2 decades.

Non-invasive tests of NAFLD can be classified by the target disease state or the type of test. Disease states of interest include the detection of hepatic steatosis (for the diagnosis of NAFLD or using steatosis improvement as treatment outcomes in early phase studies), NASH, and fibrosis. Types of tests include simple scores based on routine clinical and laboratory parameters, specific blood biomarkers, and imaging techniques. There have already been numerous reviews on non-invasive tests [14, 15]. This review focuses instead on confounding factors that affect the performance and accuracy of non-invasive tests in NAFLD. In addition, because biomarkers for NASH are less well developed and few studies have examined the confounding factors of such biomarkers, this article restricts the discussion to non-invasive tests of hepatic steatosis and fibrosis. As far as possible, we discuss confounding factors identified in original studies. For non-invasive tests without a clear analysis of confounding factors, we also discuss potential confounding factors based on the components or mechanisms of those tests.

## Non-invasive tests of hepatic steatosis

Hepatic steatosis is the defining feature of NAFLD. Current guidelines define NAFLD as the presence of ≥ 5% hepatic steatosis by histology in the absence of excess alcohol consumption [7, 16, 17]. The clinical significance of the degree of hepatic steatosis is unclear. In longitudinal studies, histological steatosis grade and controlled attenuation parameter measurement by vibration-controlled transient elastography had no or weak association with overall and liver-related mortality [18–20]. In contrast, several studies suggest that an improvement in magnetic resonance imaging proton density fat fraction (MRI-PDFF) correlate with histological improvement, defined as an improvement in the NAFLD activity score or resolution of NASH [21–23], though others have not observed such correlation [24, 25].

### Simple scores for hepatic steatosis

Simple scores for hepatic steatosis are typically derived by logistic regression or other statistical methods using clinical or laboratory factors that are associated with the presence of hepatic steatosis. The Fatty Liver Index, United States Fatty Liver Index and the Study of Health in Pomerania (SHIP) score, for example, were derived using abdominal ultrasonography as the reference standard [26–28]. Other scores used more accurate measurements of hepatic steatosis as reference standards. For instance, SteatoTest was derived using artificial intelligence against liver histology of patients with different liver diseases as the reference standard [29]. The NAFLD ridge score [30] and the Dallas Steatosis Index [31] were derived using proton-magnetic resonance spectroscopy or MRI-PDFF as the reference standard. The latter has the advantage of reflecting the general population or primary care setting, where these scores would be most applicable.

Because these scores include common clinical and laboratory parameters, they can be calculated with almost no additional costs. However, the parameters are statistical associations and do not directly measure hepatic steatosis; thus, they are also subject to the influence of confounding factors (Table 1). In particular, lipids (triglycerides and/or cholesterol in Fatty Liver Index, SteatoTest, SHIP score, NAFLD ridge score and Dallas Steatosis Index) and glycemic parameters (glucose, hemoglobin A_1c_ and/or insulin in the United States Fatty Liver Index, NAFLD liver fat score, SteatoTest, NAFLD ridge score and Dallas Steatosis Index) are affected by drug treatments. Patients receiving treatment for dyslipidemia and diabetes may have a dissociation between improvements in simple scores and improvements in hepatic steatosis. The capacity of indices to reflect longitudinal changes in intrahepatic lipid following lifestyle intervention is also diet dependent, and can be monitored with moderate precision in low-fat diets but not in low-carbohydrate diets [32]. A low fat diet alters body weight, waist circumference, triacylglycerol, and gamma-glutamyl transpeptidase, and this is reflected by changes in NAFLD-Liver Fat Score and Fatty Liver Index. However, a low-carbohydrate diet appears to affect liver metabolism and insulin sensitivity differently and changes in intrahepatic fat does not result in changes in liver fat scores. The performance of these fatty liver scores in patients receiving metabolic treatment and lifestyle modifications should be clarified in future studies.

**Table 1:** Simple scores of hepatic steatosis and potential confounding factors

Score	Components	Test performance	Potential confounding factors
Fatty liver index	BMI, waist circumference, triglycerides, GGT	AUROC 0.84, Sn 87%, Sp 64%	Triglycerides affected by lipid-lowering treatment GGT affected by alcohol consumption
United States fatty liver index	Age, waist circumference, insulin, glucose, GGT, ethnicity	AUROC 0.80, Sn 86%, Sp 88%	Insulin and glucose affected by diabetic treatment GGT affected by alcohol consumption
Hepatic steatosis index	AST/ALT ratio, BMI, sex, diabetes	AUROC 0.81, Sn 93%, Sp 92%	
NAFLD liver fat score	Metabolic syndrome, diabetes, insulin, AST, ALT	AUROC 0.86, Sn 86%, Sp 71%	Insulin affected by diabetic treatment
SteatoTest	Components of FibroTest-ActiTest (GGT, total bilirubin, alpha-2-macroglobulin, apolipoprotein A1, haptoglobin, ALT) plus BMI, cholesterol, triglycerides, glucose, age, sex	AUROC 0.79, Sn 85–100%, Sp 83–100%	Haptoglobin affected by hemolysis Bilirubin affected by hemolysis, biliary pathology and Gilbert syndrome GGT affected by alcohol consumption Cholesterol and triglycerides affected by lipid-lowering treatment Glucose affected by diabetic treatment
Study of health in pomerania score	Age, AST, ALT, waist circumference, ferritin, BMI, triglycerides, gout	AUROC 0.88	Triglycerides affected by lipid-lowering treatment Ferritin is an acute phase protein
NAFLD ridge score	ALT, HDL-cholesterol, triglycerides, hemoglobin A_1c_, white blood cell count, hypertension	AUROC 0.87, Sn 92%, Sp 90%	HDL-cholesterol and triglycerides affected by lipid-lowering treatment Hemoglobin A_1c_ affected by diabetic treatment White cell count affected by infection or inflammation
Dallas steatosis index	ALT, BMI, age, sex, triglycerides, glucose, diabetes, hypertension, ethnicity	AUROC 0.82, Sn 86%, Sp 90%	Triglycerides affected by lipid lowering treatment Glucose affected by diabetic treatment

*ALT* alanine aminotransferase, *AST* aspartate aminotransferase, *AUROC* area under the receiver-operating characteristics curve, *BMI* body mass index, *GGT* gamma-glutamyl transpeptidase, *HDL* high-density lipoprotein, *NAFLD* nonalcoholic fatty liver disease, *Sn* sensitivity, *Sp* specificity

On the other hand, other than exceptional circumstances (e.g. profound weight reduction after bariatric surgery), remission of metabolic diseases is rare. A diabetic patient would continue to be considered to have diabetes mellitus even if she manages to lose weight and reduce the dosage of anti-diabetic drugs. The scores do not take the severity of disease into account. As a result, scores including metabolic diagnoses (e.g. diabetes in Hepatic Steatosis Index, NAFLD liver fat score and Dallas Steatosis Index; metabolic syndrome in NAFLD liver fat score; and hypertension in the NAFLD ridge score and Dallas Steatosis Index) may be less suitable for the detection of improvement in hepatic steatosis over time. This phenomenon also applies to other irreversible factors such as age, sex and ethnicity.

Gamma-glutamyl transpeptidase (in the Fatty Liver Index, United States Fatty Liver Index and SteatoTest) is increased with alcohol consumption [33]. Although the diagnosis of NAFLD requires exclusion of excess alcohol consumption, the distinction between NAFLD and alcohol-related liver disease is arbitrary and mainly for research purposes. In real life, many patients have fatty liver due to both metabolic factors and alcohol, and such patients often have more severe disease and worse outcomes [34]. The performance of these simple scores in patients with and without alcohol consumption is thus of practical importance and should be clarified in future studies.

In addition, haptoglobin (in SteatoTest) is affected by hemolysis. The total bilirubin (in SteatoTest) is increased in patients with hemolysis, biliary pathology and Gilbert syndrome. White cell count (in the NAFLD ridge score) is affected by infection and hematological diseases. Ferritin (in the SHIP score) is an acute phase protein and is increased in inflammatory states.

### Imaging studies

#### Routine imaging studies

In routine clinical setting, abdominal ultrasonography is primarily used to diagnose fatty liver (Table 2). Although it is inexpensive and widely available, ultrasonography is operator dependent. It is important to report the criteria to diagnose NAFLD and the interobserver concordance in clinical research using ultrasonography. Alternatively, some studies used stored ultrasound images to define NAFLD or validate the performance of operators [35]. While ultrasonography has good accuracy to diagnose fatty liver when hepatic steatosis exceeds 30%, it is less sensitive to mild steatosis [36]. Besides, ultrasonography does not perform well in patients with morbid obesity because of poor image quality. A high riding liver and focal fatty sparing also affect the interpretation of ultrasound images.

**Table 2:** Imaging studies of hepatic steatosis and potential confounding factors

Test	Potential confounding factors
Abdominal ultrasonography	Difficult examination in obese patients and patients with high riding liver and focal fatty sparing Accuracy may be lower in older patients and patients with significant liver fibrosis Operator experience
Computed tomography	Hepatic iron content may affect liver attenuation
Controlled attenuation parameter	Failure rate higher in obese patients Accuracy may also be lower in obese patients
Proton-magnetic resonance spectroscopy or magnetic resonance imaging proton density fat faction	Hepatic iron content may affect the measurement of hepatic fat fraction, but this can be corrected during analysis

In a study of 171 patients with various causes of hepatitis from Taiwan, age, body mass index and fibrosis stage were independent factors associated with discordance between ultrasonography and liver histology in the detection of hepatic steatosis [37], but the confounding effect of age was not observed in other studies [38]. Theoretically, aging is associated with renal decline and changes in echotexture. Because one of the key features of fatty liver on ultrasonography is bright liver echotexture in relation to the kidney, aging may affect this comparison. Likewise, the liver echotexture is affected by advanced fibrosis and cirrhosis, which in turn may influence the diagnosis of fatty liver.

The attenuation values of computed tomography have inverse correlation with the degree of hepatic steatosis [39, 40]. Hepatic iron overload increases liver attenuation and may affect the determination of hepatic steatosis [41]. Because of radiation exposure, computed tomography is not primarily used for the detection of NAFLD.

#### Controlled attenuation parameter

The amplitude of ultrasound waves decreases more rapidly in a steatotic liver. This explains why deeper tissues are less clear when one uses ultrasonography to examine a patient with NAFLD. Controlled attenuation parameter (CAP) by vibration-controlled transient elastography makes use of this physical phenomenon to measure the attenuation of ultrasound waves and thereby estimates the severity of hepatic steatosis. Overall, CAP has moderate accuracy in detecting fatty liver, but there is considerable overlap of CAP values among steatosis grades [42]. Nevertheless, a recent study showed that CAP was reduced in a dose–response fashion during acetyl Co-A carboxylase inhibitor treatment for NAFLD [43]. Because vibration-controlled transient elastography is a point-of-care test, its role as a monitoring tool during NASH treatment deserves further evaluation.

Similar to ultrasonography, CAP is affected by obesity. Above all, failed examinations are more common in obese patients [44], though this problem is largely mitigated by the development of the XL probe [45]. Studies from Malaysia and Japan suggest that the accuracy of CAP for the detection of hepatic steatosis was also lower in obese patients [46, 47]. Moreover, significant liver fibrosis may affect ultrasound attenuation and lower the diagnostic performance of CAP [47].

Although food intake and active hepatitis are well-known causes of false positive liver stiffness measurement, these factors do not appear to affect CAP [48, 49]. With that said, because CAP and liver stiffness are measured simultaneously during vibration-controlled transient elastography examination, clinicians should still ask patients to fast before examination and refrain from performing vibration-controlled transient elastography in patients with risk factors of false positive results (see below).

Studies from Europe, the United States and Asia suggest that the interquartile range of CAP can serve as reliability criteria of CAP. If the interquartile range exceeds 30–40 dB/m, the accuracy of CAP measurements may be reduced [50–52]. Although another multicenter study from the United Kingdom suggests otherwise, that study only included patients suspected to have NAFLD and did not have a sufficient number of controls for comparison [53].

#### Magnetic resonance imaging

Proton-magnetic resonance spectroscopy and MRI-PDFF are highly reproducible and accurate and can be considered as the gold standard to quantify hepatic steatosis. Because the two techniques have almost identical accuracy and MRI-PDFF examines the entire liver and does not require additional sequences, the former is not the preferred MRI-based technique [54]. Although iron deposition may affect the estimation of steatosis, the overall effect is mild and can be corrected during analysis [55].

## Non-invasive tests of hepatic fibrosis

Fibrosis is undoubtedly the histological feature with the strongest correlation with liver-related morbidity and mortality [19, 56]. Portal hypertension and cirrhotic complications only develop in patients with cirrhosis [13, 57, 58]. Although hepatocellular carcinoma has been well reported in non-cirrhotic patients with NAFLD [59], cirrhosis remains one of the most important risk factors for hepatocellular carcinoma [60, 61], and the absolute incidence of hepatocellular carcinoma in the non-cirrhotic population is very low [62]. Thus, the diagnosis of fibrosis and cirrhosis in patients with NAFLD has major prognostic implications and is pivotal in selecting patients for hepatocellular carcinoma and varices surveillance.

Furthermore, because of the close association between fibrosis and clinical outcomes, regulators such as the United States Food and Drug Administration and the European Medicines Agency recognize histological fibrosis improvement with no worsening of NASH as one of the key endpoints for conditional drug approval in phase 3 NASH trials [63].

### Simple fibrosis scores

Similar to simple scores for hepatic steatosis described above, fibrosis scores were derived and validated by statistical methods using factors that were independently associated with fibrosis (Table 3). With few exceptions, liver biopsy was the reference standard for those scores. For historical reasons, most of the scores were initially tested in patients with chronic hepatitis C and subsequently validated in patients with NAFLD. Although the diagnostic accuracy is modest, these scores are inexpensive and can be performed easily at primary care setting. In one study from the United Kingdom, a referral pathway based on the use of Fibrosis-4 index followed by the Enhanced Liver Fibrosis panel increased the identification of patients with advanced fibrosis or cirrhosis by four-fold [64]. Although none of these scores is good enough to rule in advanced fibrosis, they all have respectable negative predictive values to exclude advanced fibrosis, particularly at the community level or primary care setting [65, 66]. Importantly, several studies have confirmed their roles in excluding future development of liver-related morbidity and mortality [67, 68]. Therefore, it is reasonable to apply these scores in primary care settings. Patients with low fibrosis scores can be safely monitored.

**Table 3:** Serum tests of hepatic fibrosis and potential confounding factors

Serum tests	Components	Test performance	Potential confounding factors
AST/ALT ratio	AST, ALT	AUROC 0.66–0.74, Sn 40%, Sp 80% for F3	Poor performance in patients aged ≤ 35 years
AST-to-platelet ratio index (APRI)	AST, platelet	AUROC 0.74, Sn 65%, Sp 72% for F3	Platelets may decrease in immune thrombocytopenia purpura and bone marrow diseases Platelets may increase in blood loss or myeloproliferative disease
Fibrosis-4 index	Age, AST, ALT, platelet	AUROC 0.80, Sn 65%, Sp 97% for F3	Poor performance in patients aged ≤ 35 years Low specificity in patients aged ≥ 65 years Platelets may decrease in immune thrombocytopenia purpura and bone marrow diseases Platelets may increase in blood loss or myeloproliferative disease
NAFLD fibrosis score	Age, BMI, impaired fasting glucose or diabetes, AST, ALT, platelet, albumin	AUROC 0.75–0.82, Sn 73–82%, Sp 96–98% for F3	Poor performance in patients aged ≤ 35 years Low specificity in patients aged ≥ 65 years Platelets may decrease in immune thrombocytopenia purpura and bone marrow diseases Platelets may increase in blood loss or myeloproliferative disease Albumin may decrease in chronic illnesses, malnutrition, nephrotic syndrome and protein-losing enteropathy Ethnicity
BARD score	AST, ALT, BMI, diabetes	AUROC 0.69–0.81, Sn 62%, Sp 66% for F3	BARD score appears to be less accurate in Japanese and Chinese patients, possibly due to different fat distribution at the same BMI
FibroMeter NAFLD	Body weight, prothrombin index, ALT, AST, ferritin, fasting glucose	AUROC 0.76, Sn 22%, Sp 97% for F2; AUROC 0.77, Sn 27%, Sp 95% for F3	Prothrombin index affected by anti-coagulants Ferritin is an acute phase protein Glucose is affected by anti-diabetic treatment
Enhanced liver fibrosis panel	PIIINP, hyaluronic acid, TIMP1	AUROC 0.92, Sn 88%, Sp 81% for F1; AUROC 0.98, Sn 94%, Sp 93% for F2; AUROC 0.99, Sn 100%, Sp 98% for F3	PIIINP is increased in other fibrotic diseases or bone fracture TIMP1 is increased in cancer and inflammation
FibroTest	GGT, total bilirubin, alpha-2-macroglobulin, apolipoprotein A1, haptoglobin	Non-binary AUROC for fibrosis 0.88	Haptoglobin affected by hemolysis Bilirubin affected by hemolysis, biliary pathology and Gilbert syndrome GGT affected by alcohol consumption

*ALT* alanine aminotransferase, *AST* aspartate aminotransferase, *AUROC* area under the receiver-operating characteristics curve, *BMI* body mass index, *GGT* gamma-glutamyl transpeptidase, *NAFLD* nonalcoholic fatty liver disease, *PIIINP* procollagen III amino-terminal peptide, *Sn* sensitivity, *Sp* specificity, *TIMP1* tissue inhibitor of metalloproteinases 1

Few studies specifically looked at reasons for inaccurate prediction by fibrosis scores. In a multicenter European study of 634 patients with biopsy-proven NAFLD, the aspartate aminotransferase (AST)-to-alanine aminotransferase (ALT) ratio, NAFLD fibrosis score and Fibrosis-4 index performed poorly for the diagnosis of advanced fibrosis in those aged 35 years or below [69]. In the same study, the specificity of the Fibrosis-4 index and NAFLD fibrosis score decreased to unacceptable levels in patients aged 65 years or above. This is because age is a component of these two fibrosis scores [70, 71]. On the other hand, Fibrosis-4 index and NAFLD activity score do not appear to be affected by body mass index or ALT level [72, 73]. In contrast, in our experience, AST is often higher than ALT in normal individuals with normal ALT level (unpublished results from our population cohort) [74]. One should exercise caution when interpreting scores with AST/ALT ratio as a component in patients with normal ALT.

Platelet count is a component of the AST-to-platelet ratio index, Fibrosis-4 index and NAFLD fibrosis score because thrombocytopenia is a feature of cirrhosis due to hypersplenism [70, 71, 75]. However, platelets may also decrease in immune thrombocytopenia purpura and bone marrow diseases. On the other hand, platelets may increase in myeloproliferative disease or in response to blood loss. It has been reported that the accuracy of non-invasive fibrosis scores in identifying advanced fibrosis may be reduced if the platelet count is greater than 150 × 10^9^/L [76]. The NAFLD fibrosis score also includes albumin, as protein synthesis is impaired in patients with advanced liver disease [71]. However, hypoalbuminemia may also develop in other conditions, such as in patients with chronic illnesses, malnutrition, nephrotic syndrome or protein-losing enteropathy.

In addition, the diagnostic accuracy of the non-invasive tests may be altered depending on the ethnicity. It is well known that the severity of NAFLD differs between ethnic groups. South Asians develop more metabolic complications at lower body mass indices, compared to Western populations. A study showed that the accuracy of the NAFLD fibrosis score, AST-to-platelet ratio index (APRI), FIB-4, AST/ALT ratio and BARD score is lower in the South Asian population compared to the Caucasian population [76]. Furthermore, the NAFLD fibrosis score has a lower sensitivity in patients of South Asian descent, since most of them had a lower BMI and were younger than Caucasian patients with a similar disease stage, and thus had a lower score (as BMI and age are components of the score). In contrast, another multicenter study of Southeast Asian (Malaysian and Chinese) and Caucasians showed that ethnicity did not affect the performance of the non-invasive tests performed [73].

Furthermore, studies from Japan and Hong Kong suggest that the BARD score is less accurate than what was reported initially in a Caucasian cohort [65, 77]. Although the reason for this is unclear, one possible explanation is that BARD score includes BMI, and Asian patients have different fat distribution at the same BMI.

Although FibroMeter NAFLD is calculated using a proprietary formula, the components are simple clinical and laboratory parameters: age, body weight, platelets, AST, ALT, ferritin and fasting plasma glucose [78]. Ferritin is an acute phase protein that is increased in systemic inflammation or infection, and glucose may be affected by anti-diabetic treatment.

### Specific fibrosis biomarkers

While the simple fibrosis scores are inexpensive, the components are not direct measurement of fibrogenesis or fibrinolysis and are therefore subject to various confounding effects. In contrast, there are also commercially available specific fibrosis biomarkers for the assessment of hepatic fibrosis in different liver diseases.

The Enhanced Liver Fibrosis (ELF) panel, consisting of procollagen III amino-terminal peptide (PIIINP), hyaluronic acid and tissue inhibitor of metalloproteinases 1 (TIMP1), has been validated in cross-sectional studies against liver biopsy and used alongside with liver biopsy in a number of phase 2 and 3 NASH trials [79]. In healthy people, the ELF score is higher in men and older subjects [80]. Besides, type III collagen is found in not only the liver but also many other organs together with type I collagen. Elevation of PIIINP level has been reported in bone fracture [81] and other fibrotic diseases such as burns [82], interstitial lung disease [83] and kidney disease [84]. TIMP1 is also increased in cancer [85] and inflammatory conditions such as psoriatic arthritis [86].

FibroTest comprises of gamma-glutamyl transpeptidase, total bilirubin, alpha-2-macroglobulin, apolipoprotein A1 and haptoglobin. Factors affecting GGT, haptoglobin and bilirubin levels have been described under SteatoTest and summarized in Table 3.

Pro-C3 measures the propeptide cleaved off from the intact collagen molecule and thus reflects type III collagen formation [87, 88]. It may also be increased in other fibrotic diseases, but data is scarce.

### Imaging studies

#### Ultrasound elastography

Vibration-controlled transient elastography measures the velocity of shear wave in the liver parenchyma to estimate liver stiffness [89]. It has been extensively validated against liver histology [65, 90], and correlates with clinical outcomes in longitudinal studies [91]. Although validation studies are fewer, point-shear wave elastography and two-dimensional shear wave elastography can be performed together with a regular ultrasound examination and therefore allow structural examination and hepatocellular carcinoma surveillance within the same session [92].

Pathologies that increase liver stiffness can lead to false positive diagnosis of advanced fibrosis or cirrhosis (Table 4). Grossly elevated liver stiffness has been reported in patients with acute viral hepatitis or acute exacerbation of chronic hepatitis B [93–95], though these conditions should have been excluded in the evaluation of NAFLD. Food intake also increases liver stiffness, probably through an increase in portal blood flow [96, 97]. Other well-characterized causes of spuriously high liver stiffness include congestive heart failure [98], biliary obstruction [99] and amyloidosis [100]. Solitary liver lesions such as hepatic cysts and hemangiomas have also been shown to increase liver stiffness measurement [101].

**Table 4:** Imaging studies of hepatic fibrosis and potential confounding factors

Test	Potential confounding factors
Ultrasound elastography (vibration-controlled transient elastography, point-shear wave elastography, 2-dimensional shear wave elastography)	Food intake Active hepatitis Biliary obstruction Congestive heart failure Amyloidosis Obesity and/or hepatic steatosis Inability to hold breath (COPD)
Magnetic resonance elastography	Active hepatitis Biliary obstruction Iron overload Amyloidosis Sarcoidosis Sinusoidal obstruction syndrome

In addition, high body mass index and severe hepatic steatosis have been reported to increase the false positive rate of vibration-controlled transient elastography [102–104]. However, the effect of hepatic steatosis is not easily dissected from that of obesity, and the association between hepatic steatosis and high liver stiffness has not been consistently observed in other studies [65]. Nonetheless, a recent study suggests that steatosis does not increase liver stiffness independent of fibrosis when the XL probe is used in obese patients [90].

#### Magnetic resonance imaging

Magnetic resonance elastography measures liver stiffness by a modified phase-contrast method to image the propagation of shear wave in the liver [105]. By head-to-head comparison, magnetic resonance elastography has higher applicability and accuracy than vibration-controlled transient elastography [106, 107]. Although not systematically studied, factors increasing liver stiffness described above should also affect the performance of magnetic resonance elastography. Isolated reports also suggest that liver stiffness measurement by magnetic resonance elastography is affected by iron overload, sarcoidosis and sinusoidal obstruction syndrome. We did not identify any study on the influence of food intake on liver stiffness measurement by magnetic resonance elastography, though radiologists usually advise patients to fast before MRI examinations.

Corrected T1 measurement by MRI correlates with necroinflammation and fibrosis [108, 109], and has been shown to predict liver-related events in a small study [110]. Data on the confounding factors of corrected T1 are limited.

## Conclusion

Because a substantial number of people in the community have NAFLD and only a small fraction would eventually suffer from liver-related complications, non-invasive tests are preferred as the initial assessment. Many of the available tests have high negative predictive values to exclude advanced fibrosis and future liver-related events, yet false-positive diagnoses of advanced disease are common. This review summarizes the known and potential confounding factors affecting the performance of non-invasive tests. Clinicians should interpret test results with caution when the tests are applied in patients with potential confounding factors.

## Abbreviations

ALT: Alanine aminotransferase
APRI: AST-to-platelet ratio index
AST: Aspartate aminotransferase
CAP: Controlled attenuation parameter
ELF: Enhanced liver fibrosis
MRI-PDFF: Magnetic resonance imaging proton density fat fraction
NAFL: Nonalcoholic fatty liver
NAFLD: Nonalcoholic fatty liver disease
NASH: Nonalcoholic steatohepatitis
PIIINP: Procollagen III amino-terminal peptide
Pro-C3: Neo-epitope-specific competitive enzyme-linked immunosorbent assay for PIIINP
SHIP: Study of health in pomerania
TIMP1: Tissue inhibitor of metalloproteinases 1
